# In Silico Design of Human IMPDH Inhibitors Using Pharmacophore Mapping and Molecular Docking Approaches

**DOI:** 10.1155/2015/418767

**Published:** 2015-02-15

**Authors:** Rui-Juan Li, Ya-Li Wang, Qing-He Wang, Jian Wang, Mao-Sheng Cheng

**Affiliations:** ^1^Key Laboratory of Structure-Based Drug Design & Discovery of Ministry of Education, School of Pharmaceutical Engineering, Shenyang Pharmaceutical University, Shenyang 110016, China; ^2^Institute of Medicinal Biotechnology, Chinese Academy of Medical Science and Peking Union Medical College, Beijing 10050, China

## Abstract

Inosine 5′-monophosphate dehydrogenase (IMPDH) is one of the crucial enzymes in the *de novo* biosynthesis of guanosine nucleotides. It has served as an attractive target in immunosuppressive, anticancer, antiviral, and antiparasitic therapeutic strategies. In this study, pharmacophore mapping and molecular docking approaches were employed to discover novel Homo sapiens IMPDH (hIMPDH) inhibitors. The Güner-Henry (GH) scoring method was used to evaluate the quality of generated pharmacophore hypotheses. One of the generated pharmacophore hypotheses was found to possess a GH score of 0.67. Ten potential compounds were selected from the ZINC database using a pharmacophore mapping approach and docked into the IMPDH active site. We find two hits (i.e., ZINC02090792 and ZINC00048033) that match well the optimal pharmacophore features used in this investigation, and it is found that they form interactions with key residues of IMPDH. We propose that these two hits are lead compounds for the development of novel hIMPDH inhibitors.

## 1. Introduction

Inosine 5′-monophosphate dehydrogenase (IMPDH) is a rate-limiting enzyme in the* de novo* synthesis of guanine nucleotides. It catalyzes the conversion of inosine 5′-monophosphate (IMP) to xanthosine 5′-monophosphate (XMP) [1, 2] and therefore plays an important role in the regulation of cell growth [3]. There are two isoforms of Homo sapiens IMPDH (hIMPDH), labeled types I and II, which share 84% amino acid identity. hIMPDH type I (hIMPDH1) is the main species in normal leukocytes and hIMPDH type II (hIMPDH2) predominates over hIMPDH1 in the tumor cells and activated peripheral blood lymphocytes [4–8]. Gene sequence variation in the hIMPDH2 gene may contribute to the large interindividual difference of baseline hIMPDH enzyme activity, immunosuppressive efficacy, and side effects in transplant recipients receiving mycophenolic acid [9–11]. Inhibition of hIMPDH2 has become an important strategy in the treatment of diseases related to immunosuppression, cancer, and viral and parasitic infections [12–16]. Although it has long been the belief that chemotherapy would be improved with selective inhibition of hIMPDH2, this view has recently been challenged by the surprising observation that hIMPDH1 is also an antiangiogenic target [17].

The research of hIMPDH inhibitors is of great significance in providing potentially therapeutic effects against this target for disease intervention. There are three types of hIMPDH inhibitors: (i) IMP site inhibitors that occupy the binding position of the natural substrate IMP; (ii) NAD^+^ site inhibitors that occupy the site of the NAD^+^/NADH cofactor; and (iii) allosteric site inhibitors that bind to a site remote from the IMP and NAD^+^ binding pockets. Many researchers are interested in developing NAD^+^ site inhibitors, and novel inhibitors of hIMPDH have been reported in the last decade [18]. For example, mycophenolate mofetil (MMF or Cellcept), which is a prodrug of mycophenolic acid (MPA), is an uncompetitive hIMPDH inhibitor that has been approved for the prevention of acute rejection in heart, kidney, or pancreas transplantations when used in combination with cyclosporine A [19, 20]. However, an unfavorable gastrointestinal toxicity tolerability profile limits the drug's potential for the treatment of other autoimmune disorders. To overcome the limitations of MPA, Vertex developed a series of phenyl-oxazole urea hIMPDH inhibitors using structure-based drug design and high-throughput screening, among which VX-497 has been in phase II development for the hepatitis C virus (HCV) infection [21]. In addition, tiazofurin has been found to possess both antiviral and antiproliferative activities [22, 23]. Several compounds, such as quinolones [24, 25], amides [26], and indoles [27, 28], have been reported to possess potent hIMPDH inhibition activities. However, safety and selectivity are still deficient, and there is a continuing effort to develop novel hIMPDH inhibitors.

The pharmacophore model can be used to elucidate how diverse ligands bind to receptor sites and it can predict potential chemical interactions between ligands and a receptor. In addition, this model can be used to discover potent inhibitors of the target protein from selected database [29–31]. In this study, common feature pharmacophore modeling was used to uncover novel hIMPDH inhibitors from the ZINC database. Structure-based docking was then performed to analyze the binding modes and affinities of the identified compounds that show promise as hIMPDH inhibitors. Finally, interactions between IMPDH and the potential inhibitors were described in detail, with the aim to design novel drug candidates of hIMPDH.

## 2. Methods

The common feature pharmacophore model was generated using the Common Feature Pharmacophore Generation protocol in the Discovery Studio 3.0 software program (DS 3.0). Database screening was implemented using the Ligand Profiler Protocol in DS 3.0 [32]. Docking studies were performed with the glide module in the Schrödinger 2014 software program [33].

### 2.1. Pharmacophore Generation and Validation

#### 2.1.1. Ligand Preparation

**Figure 1:**
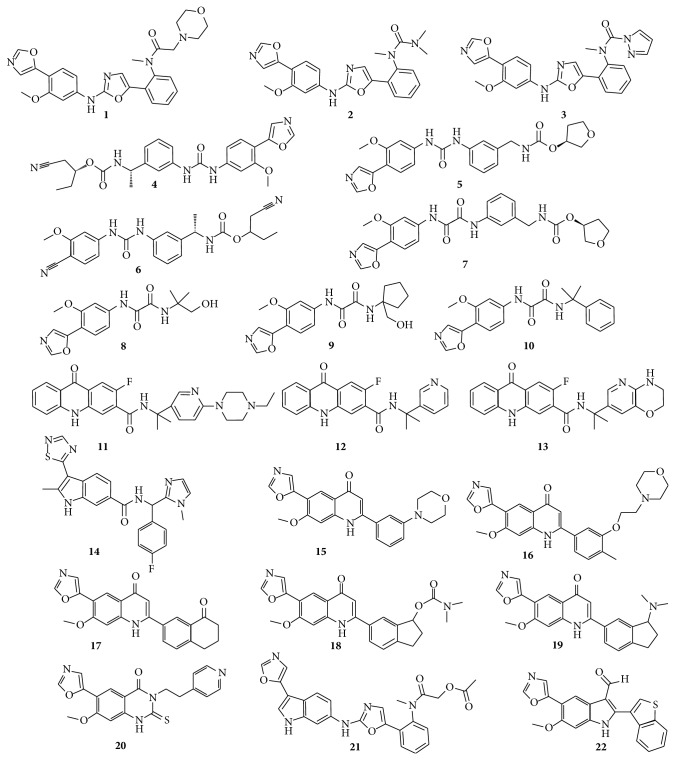
Structures of compounds** 1**–**22** in the training set for the development of pharmacophore model.

A set of ligands occupying the hIMPDH NAD^+^ site with known inhibitory activities were collected from the literature to establish a common feature pharmacophore. All of the structures were constructed in DS 3.0. The most important step in pharmacophore modeling is the selection of suitable inhibitors that constitute the training set. For this purpose, 22 active hIMPDH inhibitors with diverse scaffolds [24, 27, 34–42] (Figure 1) were selected as the training set and generated using parameters from the CHARMm force field. All of the structures were minimized using the Steepest Descent algorithm, followed by the Conjugate Gradient and Adopted Basis Newton-Raphson algorithms, with convergence gradient values of 0.1 kcal·mol^−1^, 0.01 kcal·mol^−1^ and 0.001 kcal·mol^−1^, respectively. A multiconformer database was generated using the poling algorithm with an energy threshold of 20 kcal·mol^−1^ and a maximum of 255 conformers per molecule.

#### 2.1.2. Pharmacophore Generation

Pharmacophore model generation was performed using the multiconformer database described above, with activity values listed in Table 1. For the most potent inhibitors (Compounds** 1**,** 3**,** 4**,** 5**,** 6**,** 12**,** 15**,** 16**,** 17**,** 18**,** 19**,** 20**, and** 21**), the Principal and MaxOmitFeat values were set to 2 and 0, respectively. For the moderate inhibitors (Compounds** 2**,** 7**,** 8**,** 9**,** 10**,** 11**,** 13**,** 14**, and** 22**), the Principal and MaxOmitFeat values were both assigned values of 1. The H-bond acceptor (A), H-bond donor (D), hydrophobic aliphatic (H), and hydrophobic aromatic (Z) features were selected based on the chemical features of the ligands in the training set using the Feature Mapping protocol in DS 3.0. The value of Maximum Pharmacophore was set to 10. The default Minimum Interfeature Distance value of 2.97 Å was changed to 2.0 Å so that the chemical features located close to each other would be considered when generating the pharmacophore. The minimum features were set to 4, and the maximum features were set to 7. Default values were used for all other parameters. From this approach, ten pharmacophore hypotheses were successfully generated. These models were validated with both the training set and the decoy set.

#### 2.1.3. Pharmacophore Validation

The training set compounds were aligned to the ten generated pharmacophore hypotheses using the Ligand Profiler Protocol in DS 3.0. The Maximum Omitted Features option was set to −1, and the Scale Fit Values were set to false. Default values were used for all other parameters. To confirm the quality of these models, the ten generated pharmacophore hypotheses were assessed by rank scores and fit values. Subsequently, some poor models were rejected and the remaining pharmacophore models were assessed using another method. In general, pharmacophore models are used as 3D queries to search databases to discover novel and potent lead compounds. A validation of the generated pharmacophore model should be performed to determine whether the model is able to accurately differentiate between active and inactive compounds. The Güner-Henry (GH) scoring method was used to validate the pharmacophore hypotheses [43, 44]. This method quantifies the merit of the generated model by retrieving the active compounds from a database containing known active and inactive molecules. A decoy set containing 729 molecules was constructed to validate the generated pharmacophore. Of these 729 molecules, 29 molecules were known inhibitors of hIMPDH, whereas the other 700 molecules were obtained from the ZINC database using the Find Similar protocol in DS 3.0. The GH score method has been successfully applied to quantify the selectivity of the pharmacophore model and to discover activities from a decoy database. The GH score was calculated using the following formulae:

(1) $$\begin{array}{c} Y\%=\frac{H_{a}}{T_{a}}\times 100\%\\ H_{t}\%=\frac{H_{a}}{H_{t}}\times 100\%\\ E=\frac{H_{a}/H_{t}}{T_{a}/T}\\ \text{GH}=\frac{H_{a}\left(3H_{a}+H_{t}\right)}{4H_{t}T_{a}}\left(1-\frac{H_{t}-H_{a}}{T-T_{a}}\right), \end{array}$$

where *H*_*a*_ is the number of active hits, *T*_*a*_ is the total number of actives in the decoy set, *H*_*t*_ is the total number of hits including actives and decoy molecules, *T* is the total number of molecules in the decoy set, *Y*% is the percentage of known active compounds obtained from the decoy set, *H*_*t*_% is the percentage of known actives in the hits list, *E* is the enrichment factor, and GH is the Güner-Henry score. The GH score ranging from 0.6 to 1 would indicate an optimal pharmacophore model.

### 2.2. Database Searching

Virtual screening of a chemical database often leads to the discovery of novel and potential lead compounds for further development. The optimal pharmacophore model (hypo-07) was used as the 3D query for screening the ZINC database, which contains 325,881 drug-like molecules. All screening experiments were performed using the Ligand Profiler Protocol in DS 3.0. During the screening process, we performed the virtual screening using the Best/Flexible conformational procedure, with a maximum of 255 conformations generated. The Maximum Omitted Features option was set to −1, and the Scale Fit Values were set to false. Molecules that possessed all the desired pharmacophore features were considered to be hit compounds. The so-called “Lipinski's rule of five” was not applied to selected compounds because we wanted to prevent the removal of potential inhibitors. Those molecules that successfully passed these initial tests were selected for subsequent molecular docking analysis.

### 2.3. Molecular Docking

Docking study is a necessary step for picking out potential hits in virtual screening [45]. To investigate the detailed interactions between the virtual hits and IMPDH, the glide module in the Schrödinger software program was used to perform docking studies.

The 3D structure of IMPDH in complex with IMP and MPA (PDB ID: 1JR1) has been determined by X-ray diffraction. MPA inhibits IMPDH by acting as a replacement for the nicotinamide portion of the nicotinamide adenine dinucleotide cofactor (NAD^+^) and a catalytic water molecule. Our primary aim was to obtain NAD^+^ site inhibitors; thus, the 1JR1 crystal structure was used for docking studies and the position of MPA was selected as the active site. The protein was prepared with the Protein Preparation Wizard (PrepWizard) in the Schrödinger software program. The location of MPA in the 1JR1 crystal structure was used to define the size and center of the receptor grid. An active site of 5 Å was set around the MPA ligand. To prepare the protein, MPA was deleted, IMP was retained, hydrogen atoms were added, water molecules beyond 5 Å from the groups were deleted, and the bond orders of the protein were adjusted and minimized up to 0.30 Å root mean square deviation (RMSD). Then, the original conformation of the MPA was docked into the NAD^+^ site of IMPDH using both rigid and flexible methods to validate the docking procedure. When the RMSD value ranges between 0 and 2, the program can be used for docking studies of other ligands. The ideal case is when the RMSD value is below 0.5. Finally, the extra precision (XP) mode and other default parameters of the glide module were used for the docking studies [46]. The compounds of the training set and the hit compounds were docked into the active site of IMPDH using the flexible docking strategy to predict potential inhibitors of IMPDH.

## 3. Results

### 3.1. Generation and Validation of the Common Feature Pharmacophore Model of hIMPDH Inhibitors

A pharmacophore, which is known as a powerful tool to identify novel compounds with good biological activity, can be established via a ligand-based method. The common feature pharmacophore generation protocol in DS 3.0 was employed with the training set compounds. As suggested by the Feature Mapping protocol, pharmacophore models were generated using the following features: H-bond acceptor (A), H-bond donor (D), hydrophobic_aliphatic group (H), and hydrophobic_aromatic group (Z). Ten pharmacophore hypotheses were obtained for further investigation based on the training set molecules. All ten pharmacophore hypotheses contain the above four chemical features. Statistical parameters of the generated pharmacophore models are listed in Table 2. Rank scores of the ten models range from 215.59 kcal·mol^−1^ to 229.50 kcal·mol^−1^. Direct and partial hit values of 1 and 0 indicate that the ligands are well mapped onto all of the chemical features of the model (i.e., there are no missing features). The max fit of all the hypotheses is 4. Clearly, the fit values of hypotheses 03, 04, 07, and 08 (i.e., hypo-03, -04, -07, -08) are higher than those of hypo-01, -02, -05, -06, -09, and -10. For this reason, the former four hypotheses were selected for further investigation (Table 3). However, on the basis of rank scores and fit values alone, we could not determine which pharmacophore hypothesis is the best model. Therefore, the Güner-Henry (GH) scoring method was used to identify the best pharmacophore model. A decoy database, which was used for pharmacophore identification and validation, was constructed to include 20 independent active hIMPDH inhibitors, 9 inactive hIMPDH inhibitors, and 700 inactive ligands from the ZINC database. Notably, all of the 20 active molecules were successfully identified by hypotheses 03, 04, and 07, and the number of total hits was 66, 75, and 35 for each hypothesis, respectively. Other statistical parameters, such as yield of actives, hit rate of actives, enrichment factor, and GH score, are presented in Table 4. The GH score is one of the standards used to assess the quality of generated models. In this regard, hypothesis 07 (hypo-07) demonstrates the highest GH score value of 0.67, which indicates a strong capability to choose the active rather than inactive molecules from the database.

**Figure 2:**
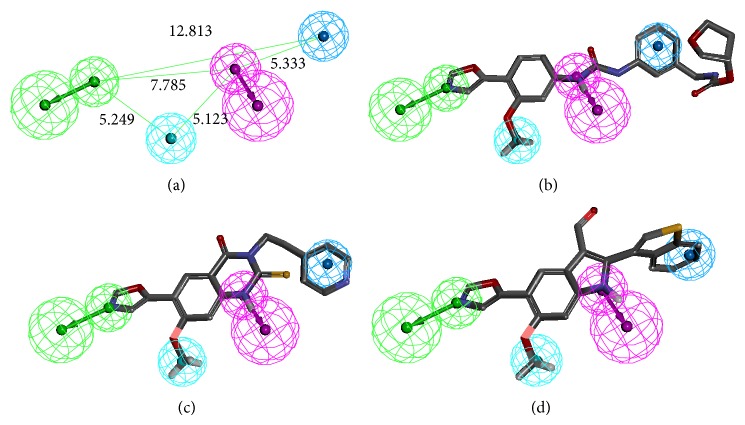
(a) 3D spatial relationship and geometric parameters of hypo-07. Pharmacophore features colored as follows: H-bond acceptor (green), H-bond donor (magenta), hydrophobic_aliphatic group (cyan) and hydrophobic_aromatic group (sky blue). (b) Hypo-07 mapping with active compound** 5**. (c) Hypo-07 mapping with active compound** 20**. (d) Hypo-07 mapping with moderate active compound** 22**.

The 3D spatial relationship and geometric parameters of hypo-07 are shown in Figure 2(a). Figures 2(b)–2(d) describe the alignment of hypo-07 with three different inhibitors (i.e., compounds** 5** (*K*_*i*_ = 7 nM),** 20** (IC_50_ = 13 nM), and** 22** (IC_50_ = 30 nM), resp.). Clearly, the four pharmacophore features map very well onto compounds** 5** and** 20**, which have previously been shown to be potent inhibitors. In addition, other potent inhibitors such as compounds** 1**,** 3**,** 4**,** 6**,** 15**,** 16**,** 17**,** 18**,** 19**,** 20**, and** 21**, but not compound** 12**, present good hit values (Table 3). For the moderately active compound** 22**, the H-bond acceptor and hydrophobic_aliphatic pharmacophore features could match with the ligand but the H-bond donor and the hydrophobic_aromatic features did not. These results reveal that a selected pharmacophore model might be capable of predicting the activity of compounds. Consequently, hypo-07 was applied for further studies to identify novel inhibitors.

### 3.2. Database Screening

The best pharmacophore model (i.e., hypo-07) was used to search the ZINC database, which contains 325,881 compounds, for new hIMPDH inhibitor candidates. Among the 325,881 compounds, 1566 compounds passed the initial screening and were mapped onto the pharmacophore model, which included some compounds that are structurally similar to existing hIMPDH inhibitors and some novel scaffolds. Sixty-nine hits were selected based on the fit value. All of these compounds have common features that can align to the required pharmacophore sites of hypo-07. The 69 hit compounds were exported as an.sdf file and subjected to further analysis using molecular docking to avoid possible false positive hits from the virtual screening process.

### 3.3. Molecular Docking Studies of hIMPDH Inhibitors and Hit Compounds

#### 3.3.1. Docking Validation

**Figure 3:**
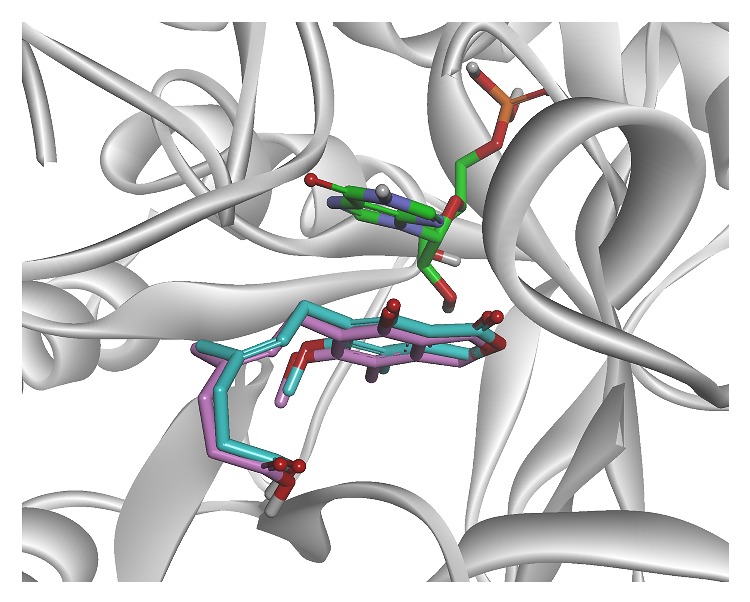
The alignment of redocked (pink) and cocrystallized ligand (cyan) in the active site of IMPDH. The green ligand is IMP.

In this study, the validation of the docking procedure was performed by redocking cocrystallized MPA into the active site of IMPDH using both the rigid and flexible methods of the glide module. We found that the redocked MPA reproduced the binding pose with glide scores of −6.65 kcal·mol^−1^ and −6.59 kcal·mol^−1^, respectively. The RMSD of the cocrystallized and experimental poses was analyzed, and the values of the two methods were 0.54 Å and 0.43 Å, respectively. These results showed that docking simulations reproduced the crystal complexes very well. The alignment of the cocrystallized ligand (green) and redocked ligand (pink) is shown in Figure 3. The glide XP program was suitable for further studying the binding pose of the novel hits.

#### 3.3.2. The Docking Results

The nonbond interactions were examined using the similarly labeled tool in the Discovery Studio 4.0 Visualizer software program; various interactions between the inhibitors and IMPDH were examined, including traditional hydrogen bonding interactions, carbon-hydrogen bonding interactions, pi-donor hydrogen bonding interactions, and hydrophobic interactions. According to the docking results, we can see that the interface points of IMPDH include interactions from Pro69, Met70, Asp71, His92, His93, Asn94, Cys95, Ala249, Gly251, Thr252, His253, Asp256, Arg259, Asp274, Ser275, Gln277, Asn303, Arg322, Gly324, Met325, Gly326, Cys327, Gly328, Cys331, Ile332, Thr333, Gln334, Asp364, Met414, Gly415, Ala419, Met420, and Gln441. On the basis of these results, some active sites and residues were identified from the IMPDH complex.

First, the docking results of MPA and compound** 5** (i.e., VX-497) of the training set are described. On one hand, research on the known active ligands can help us to identify the suitable binding modes of the inhibitors in the NAD^+^ site of IMPDH. On the other hand, the analysis of interactions between VX-497 and IMPDH can rationalize the pharmacophore model from another aspect.

**Figure 4:**
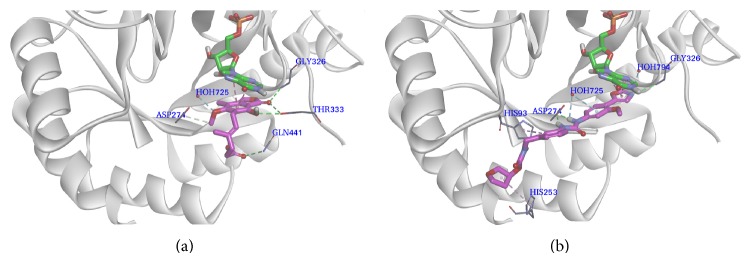
Binding patterns of potent inhibitors with IMPDH. The secondary structure of IMPDH is shown as a white rainbow. Residues in the active site are shown in gray lines and labelled as amino acid names. IMP is shown in green stick. The H-bonding interactions are shown using green dotted lines, weak H-bonding interaction is shown using pale-cyan dotted lines, and hydrophobic interactions are shown using pink dotted lines. (a) The binding mode of cocrystallized ligand (MPA, magenta) in the active site of IMPDH. (b) The binding mode of compound** 5** (VX-497, magenta) in the active site of IMPDH.

MPA exhibits various interactions with the amino acid residues of the active site, as shown in Figure 4(a). The interactions between MPA and IMPDH active site residues were identical with the cases reported by Sintchak and Nimmesgern [47]. The results suggest that the carbonyl group of the lactone ring bonds to IMPDH with its oxygen atom to form hydrogen bonding interactions with the backbone NH moiety of Gly326 and the OH group of Thr333. At the same time, the oxygen atom of the hydroxyl group of MPA forms a hydrogen bonding interaction with the OH group of Thr333. The carbonyl group of COOH forms a hydrogen bonding interaction with the NH moiety of Gln441. The oxygen atom (O3) of the methoxy group forms a weak water-hydrogen bonding interaction with H_2_O725 and the carbon atom (C8) of the methoxy group is involved in a carbon-hydrogen bonding interaction with Asp274. IMP, which is another ligand of IMPDH, was retained during docking studies because its purine ring forms important *π*-*π* hydrophobic interactions with MPA.

The binding models between VX-497 and IMPDH active site residues are similar to most of the interactions reported by Sintchak and Nimmesgern [47]. The docking analysis demonstrates that the oxazole of VX-497 bonds to IMPDH via its nitrogen atom to form a hydrogen bonding interaction with the NH moiety of Gly326. The same nitrogen atom also forms a water-hydrogen bonding interaction with H_2_O794. The benzene ring connected with oxazole makes a *π*-donor hydrogen bonding interaction with H_2_O725. The additional hydrogen bonding interactions are formed between the NH moiety of the urea group and Asp274. Notably, the nitrogen atom of oxazole and the NH moiety of the urea group serve as a H-bond acceptor and a H-bond donor, respectively, which is identical to the analogous features of the best pharmacophore model. In addition, another benzene ring of VX-497 forms a *π*-*π* hydrophobic interaction with His93, corresponding to the aromatic hydrophobic feature of the pharmacophore model. At the same time, the tetrahydrofuran ring of the inhibitor forms a *π*-alkyl hydrophobic interaction with His253. The *π*-*π* hydrophobic interactions between the inhibitor and the purine ring of IMP are also formed (Figure 4(b)).

From the above analyses, we believe that the role of Gly326 is to serve as a H-bond donor, which is necessary for potent inhibitors. An interaction between Thr333 and the ligand is also important. In addition, the critical hydrogen bonding interaction between the urea NH moiety and Asp274 contributes to the high potency observed for VX-497. Some hydrophobic interactions also play important roles in improving the activities of compounds.

The sixty-nine hit compounds that were predicted to be positive using the pharmacophore screening procedure were subjected to molecular docking studies. The potential compounds were selected according to the glide scores and their interactions with amino acid residues. Notably, ten hit compounds (see Figure S1 in Supplementary Material available online at http://dx.doi.org/10.1155/2015/418767) were found to have good glide scores compared to MPA and VX-497 (Table 5). Here, the binding modes of the two identified compounds with the highest glide scores from the ZINC database are described. They are ZINC02090792 and ZINC00048033, which are used for subsequent characterization as potential inhibitors.

**Figure 5:**
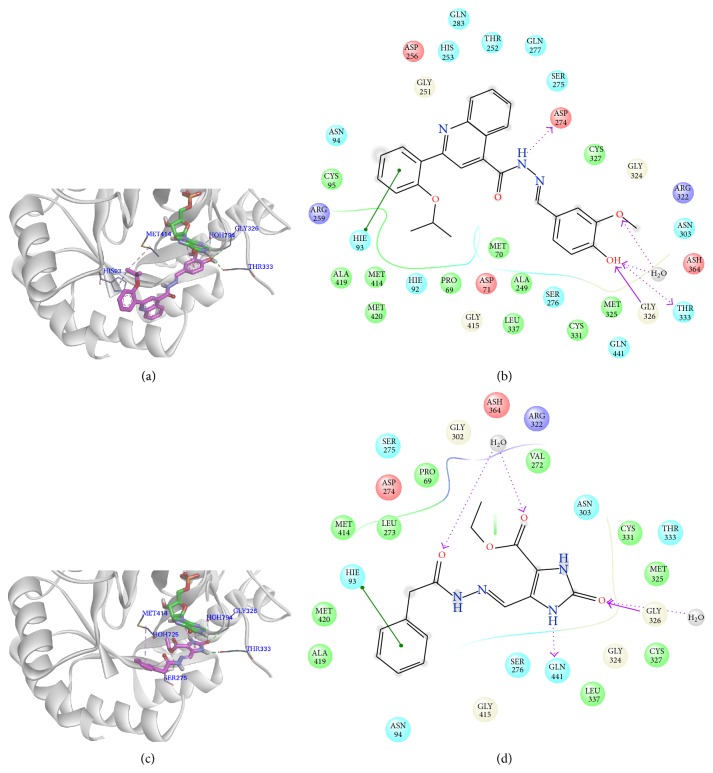
Binding mode and 2D interaction map of hit compounds. (a) The binding mode of ZINC02090792 in the active site of IMPDH. (b) The 2D interaction map of ZINC02090792 with the active site of IMPDH. (c) The binding mode of ZINC00048033 in the active site of IMPDH. (d) The 2D interaction map of ZINC00048033 with the active site of IMPDH.

The top virtual hit compound, ZINC02090792, shows similar binding interactions to MPA and VX-497 (Figure 5(a)). The oxygen atom of the hydroxyl group serves as a H-bond acceptor to form a hydrogen bonding interaction with the backbone NH moiety of Gly326, and the hydroxyl group serves as a H-bond donor to form a hydrogen bonding interaction with Thr333. At the same time, both the hydroxyl and methoxy groups of ZINC02090792 form water-hydrogen bonding interactions with H_2_O794. From the 2D interaction map (Figure 5(b)) of ZINC02090792 in the active site of IMPDH, it is observed that NH serves as a H-bond donor engaging in an interaction with Asp274. One carbon atom (C3) belonging to the methyl group of ZINC02090792 forms a *π*-alkyl hydrophobic interaction with His93, and it also makes an alkyl-alkyl hydrophobic interaction with Met414. In addition, the benzene ring connected with the hydroxyl and methoxy groups forms *π*-*π* hydrophobic interactions with the purine ring of IMP.

ZINC00048033 is another hit compound obtained using pharmacophore screening and docking validation. The interactions between ZINC00048033 and IMPDH are presented in Figures 5(c) and 5(d). The carbonyl group of the 1*H*-imidazole-2-(3*H*)-one ring forms a hydrogen bonding interaction with the backbone NH moiety of Gly326, and it also makes a water-hydrogen bonding interaction with H_2_O794. Additionally, the NH group of 1*H*-imidazole-2-(3*H*)-one ring serves as a H-bond donor to form a hydrogen bonding interaction with Thr333. The carbonyl group that connects with the ethoxyl group forms a water-hydrogen interaction with H_2_O725. One carbon atom (C2) of ZINC00048033 engages in a carbon-hydrogen bonding interaction with Ser275. At the same time, a *π*-alkyl hydrophobic interaction between the benzene ring of ZINC00048033 and Met414 is observed. Similar to an active inhibitor of IMPDH, the 1*H*-imidazole-2-(3*H*)-one ring of ZINC00048033 forms *π*-*π* hydrophobic interactions with the purine ring of IMP.

According to the above results, ZINC02090792 and ZINC00048033 form interactions with some key residues of IMPDH (i.e., Gly326, Thr333, and Asp274) in ways that are similar to the cocrystallized ligand MPA and the most potent ligand VX-497. Superimpositions of MPA on ZINC02090792 and ZINC00048033 bound to the active site are shown in Figure S2. Despite some changes in the interactions between active site residues and inhibitors (MPA, VX-497, ZINC02090792, and ZINC00048033), the conformations of these active site residues are virtually unchanged between the crystal structures of MPA or other three inhibitors bound to the active site of IMPDH. Consequently, ZINC02090792 and ZINC00048033 serve as lead compounds for developing novel hIMPDH inhibitors.

## 4. Conclusions

We have established a ligand-based pharmacophore model followed by virtual screening and molecular docking studies to discover novel hIMPDH inhibitors. The common feature pharmacophore models were generated using a training set that includes 22 active hIMPDH inhibitors. Ten hypotheses were obtained from this analysis, which all consisted of four features, including one hydrogen-bond acceptor, one hydrogen-bond donor, one hydrophobic_aliphatic group, and one hydrophobic_aromatic group. To confirm the quality of the pharmacophore models, a decoy test set was constructed and the Güner-Henry (GH) scoring method was used. The hypo-07 model was found to possess the highest GH score value (0.67). This result reveals that hypo-07 is an optimal model to discriminate between active and inactive molecules within the database. The hypo-07 model was further used to screen the ZINC database to identify hIMPDH inhibitors, and sixty-nine potential candidates were selected. All the compounds can align to the required pharmacophore features of hypo-07. In addition, molecular docking was performed using the glide module to investigate the interactions between the potential candidates and IMPDH. From the docking studies, ten compounds were found to have higher glide scores than known active inhibitors. The top two hit compounds for IMPDH were ZINC02090792 and ZINC00048033, with corresponding glide scores of −7.80 kcal·mol^−1^ and −7.58 kcal·mol^−1^, respectively. Furthermore, the detailed interactions between the hit compounds and IMPDH were analyzed. ZINC02090792 forms hydrogen bonding interactions with Gly326, Thr333, Asp274, and H_2_O794. It also preserves a *π*-*π* hydrophobic interaction with His93 and an alkyl-alkyl hydrophobic interaction with Met414. ZINC00048033 engages in hydrogen bonding interactions with Gly326, Thr333, H_2_O725, and H_2_O794. At the same time, it forms a carbon-hydrogen bonding interaction with Ser275 and a *π*-alkyl hydrophobic interaction with Met414. The two potential hit compounds both form *π*-*π* hydrophobic interactions with the purine ring of IMP. In conclusion, the identified hits serve as lead compounds for developing potential hIMPDH inhibitors. Derivatives of these two hits have been synthesized and an evaluation of their biological activity is now in progress.

## Supplementary Material

3D structures of all ten proposed inhibitors mapping onto the ligand-based pharmacophore model are shown in Figure S1. Close-up of superimposition between MPA and the two screened inhibitors are shown in Figure S2.

## Figures and Tables

**Table 1:** Bioactivity of compounds in the training set from literature.

Compound	*K*_*i*_ [nM]	IC_50_ [nM]	Reference
**1**		12	[34]
**2**		15	[34]
**3**		11	[34]
**4**	6		[35]
**5**	7		[36]
**6**	6		[37]
**7**		30	[38]
**8**		19	[38]
**9**		18	[38]
**10**		30	[38]
**11**		17	[39]
**12**		11	[39]
**13**		17	[39]
**14**		19	[40]
**15**		5	[24]
**16**		<5	[24]
**17**		<10	[24]
**18**		11	[24]
**19**		6	[24]
**20**		13	[41]
**21**		7	[42]
**22**		30	[27]

**Table 2:** Top ten pharmacophore hypotheses generated by IMPDH inhibitors.

Hypo	Features	Rank	Direct hit^a^	Partial hit^b^	Max fit
01	ZHDA	229.50	1111111111111101111111	0000000000000010000000	4
02	ZHDA	229.42	1111111111111111111111	0000000000000000000000	4
03	ZHDA	226.70	1111111111111111111111	0000000000000000000000	4
04	ZHDA	225.26	1111110111111111111111	0000001000000000000000	4
05	ZHDA	223.74	1111110011111111111111	0000001100000000000000	4
06	ZHDA	219.08	1111110011111111111111	0000001100000000000000	4
07	ZHDA	218.00	1111110011111111111111	0000001100000000000000	4
08	ZHDA	218.10	1011111111011111111110	0100000000100000000001	4
09	ZHDA	216.52	1011111011011110111111	0100000100100001000000	4
10	ZHDA	215.59	1111110001110111111111	0000001110001000000000	4

^a^Direct hit indicates whether “1” or not “0” a molecule in the training set mapped every pharmacophore feature in the hypothesis. ^b^Partial hit connotes whether “1” or not “0” a particular molecule in the training set mapped all but one pharmacophore feature in the hypothesis.

**Table 3:** The hit values of compounds in the training set mapping to hypotheses 03, 04, 07, and 08.

Comp.	Hypo-03	Hypo-04	Hypo-07	Hypo-08
**1**	3.208	3.265	3.288	3.156
**2**	3.165	3.303	3.061	3.880
**3**	3.665	3.390	3.502	3.775
**4**	3.980	3.980	4.000	3.887
**5**	3.999	3.999	3.988	3.784
**6**	3.749	3.660	3.605	3.583
**7**	3.210	3.408	3.362	3.528
**8**	2.992	2.980	2.997	3.029
**9**	2.990	2.994	2.977	3.648
**10**	3.578	3.369	3.661	3.078
**11**	2.669	2.776	2.780	2.865
**12**	2.658	2.7281	2.764	2.830
**13**	2.436	2.750	2.825	3.146
**14**	2.889	3.161	2.898	2.255
**15**	3.189	3.086	3.428	3.999
**16**	3.086	3.133	3.444	3.262
**17**	3.160	3.031	3.338	3.475
**18**	3.115	3.032	3.277	3.652
**19**	3.100	3.099	3.261	3.745
**20**	3.868	3.796	3.771	2.998
**21**	3.613	3.239	3.343	3.307
**22**	2.987	3.046	3.096	2.914

**Table 4:** The evaluation of ligand-based pharmacophore model using the Güner-Henry scoring method.

	Hypo-03	Hypo-04	Hypo-07	Hypo-08
Total number of molecules in the decoy set (*T*)	729	729	729	729
Total number of actives in the decoy set (*T*_*a*_)	20	20	20	20
Active hits (*H*_*a*_)	20	20	20	15
Total hits (*H*_*t*_)	66	75	35	54
Yield of actives (*Y*%)	100%	100%	100%	75%
Hit rate of actives (*H*_*t*_%)	30.30%	26.67%	57.14%	27.78%
Enrichment factor (*E*)	11.05	9.72	20.83	10.13
Goodness of hit score (GH)	0.45	0.41	0.67	0.50

GH: Güner-Henry.

**Table 5:** The glide score of hit compounds compared with MPA and VX-497.

Comp.	Glide XP Gscore (kcal·mol^−1^)	Glide energy
(1) ZINC02090792	−7.80	−59.779
(2) ZINC00048033	−7.58	−59.395
(3) ZINC00822338	−7.57	−64.007
(4) ZINC08714541	−7.17	−58.716
(5) ZINC06662648	−7.06	−60.263
(6) ZINC00686714	−6.98	−64.254
(7) ZINC00648305	−6.95	−55.144
(8) ZINC02081544	−6.93	−62.507
(9) ZINC00668789	−6.90	−57.154
(10) ZINC02776094	−6.88	−55.596
(11) MPA	−6.59	−54.289
(12) VX497	−7.56	−60.157
