# Supplementary material for: In Silico Design of Human IMPDH Inhibitors Using Pharmacophore Mapping and Molecular Docking Approaches
Source: Comput Math Methods Med. 2015 Feb 15;2015:418767. doi: 10.1155/2015/418767 (PMC4345060; doi:10.1155/2015/418767)
Supplement: Supplementary file 1 — 3D structures of all ten proposed inhibitors mapping onto the ligand-based pharmacophore model are shown in Figure S1. Close-up of superimposition between MPA and the two screened inhibitors are shown in Figure S2. [file 418767.f1.pdf]

Supplementary Materials

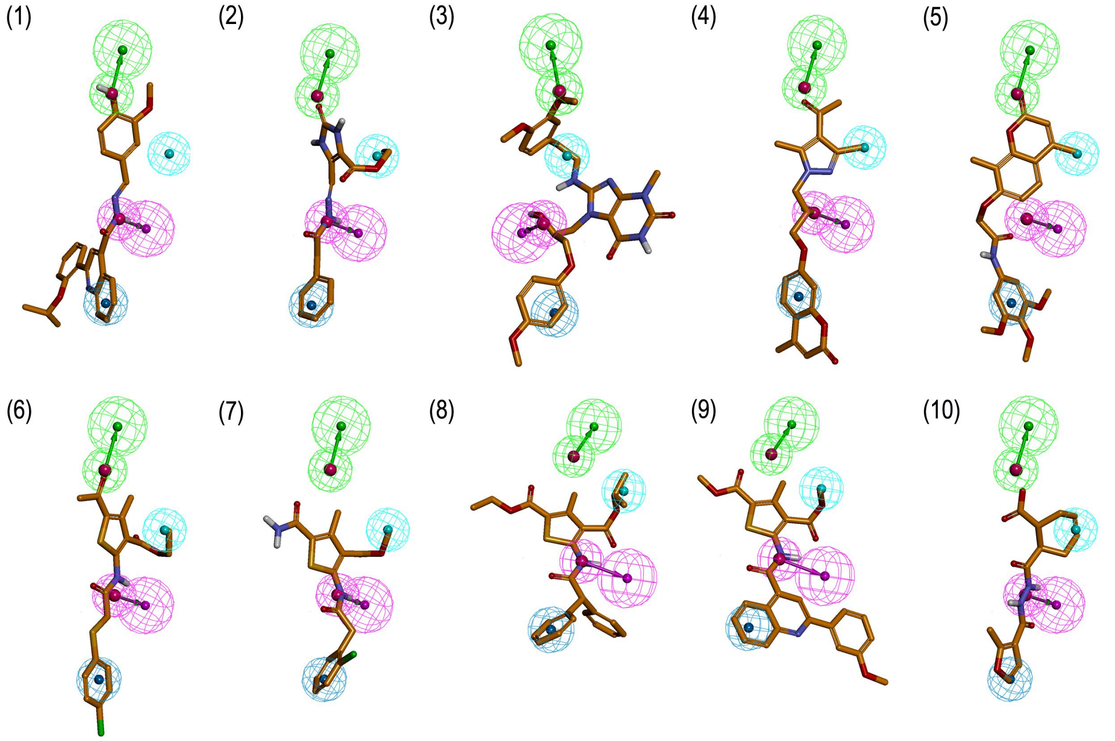

Figure S1. 3D structures of all ten proposed inhibitors mapping onto the ligand-based pharmacophore model.

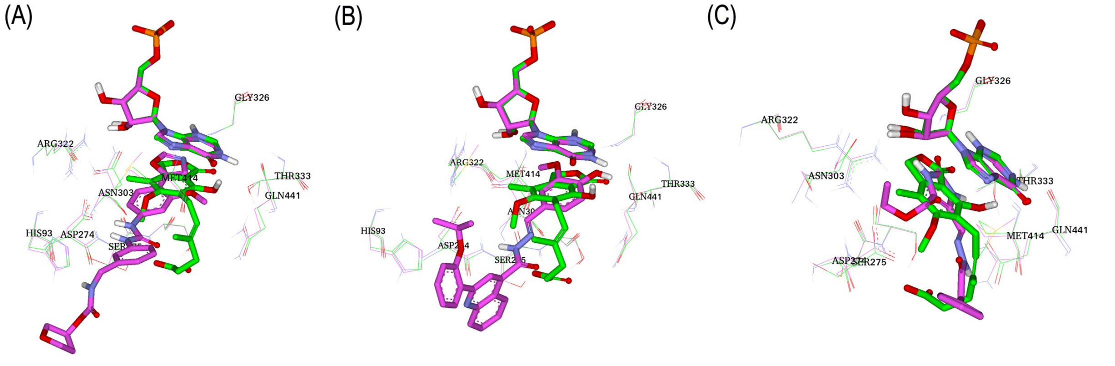

Figure S2. Close-up of superimposition between 1JR1 crystal structure (green) and docked structures of IMPDH in complex with inhibitors (magenta). For clarity, side chains that form this position of the inhibitor-binding pocket have been labeled. (A) A superimposition of MAP and VX-497. (B) A superimposition of MAP and

ZINC02090792. (C) A superimposition of MAP and ZINC00048033.
